# Special Issue “The Next Generation of Upper Gastrointestinal Endoscopy”

**DOI:** 10.3390/diagnostics12010152

**Published:** 2022-01-09

**Authors:** Hiroki Kurumi, Hajime Isomoto

**Affiliations:** Division of Gastroenterology and Nephrology, Department of Multidisciplinary Internal Medicine, Faculty of Medicine, Tottori University, 36-1 Nishicho, Yonago 683-8504, Japan; kurumi_1022_1107@yahoo.co.jp

Upper gastrointestinal endoscopy is now widely used as a first-line procedure to investigate upper gastrointestinal symptoms in most countries around the world. Since Bozzini developed the first method of examining bodily orifices in 1805 and Desormeaux developed the first effective open tube endoscope in 1853 [1], upper gastrointestinal endoscopy has rapidly evolved through various transitional periods such as the development of image enhanced endoscopy (IEE) and the application of artificial intelligence (AI). In this Special Issue, eight articles on the latest findings in upper gastrointestinal endoscopy are presented.

Ikebuchi et al. developed a new mouthpiece to reduce pain associated with endoscopy, and evaluated its effectiveness using the visual analog scale (VAS) [2]. Their study showed that the new mouthpiece had a lower VAS during endoscopy compared to the conventional mouthpiece (1.7 ± 1.5 vs 2.5 ± 2.4 cm, *p* = 0.06), but the difference was not significant. It is also worth noting that this mouthpiece is designed to reduce the gag reflex. The COVID-19 pandemic has had a major impact on upper gastrointestinal endoscopy practices worldwide [3,4]. In upper gastrointestinal endoscopy, aerosol-generating procedures are performed at close range, raising concerns about the inadvertent exposure of healthcare workers and the possibility of infection [5,6]. This new mouthpiece may be able to reduce the generation of aerosols by suppressing the vomiting reflex.

Fujiwara et al. also responded quickly to ensure the safety of endoscopists from the unprecedented situation of the COVID-19 pandemic. They developed a patient-covering negative-pressure box system (EB) and investigated whether it could reduce the exposure of aerosols and droplets to the endoscopist during upper gastrointestinal endoscopy [7]. They evaluated the amount of aerosol dispersed outside the EB during upper gastrointestinal endoscopy using a handheld optical particle counter, and the amount of droplets adhering to the endoscopist was evaluated by the ATP contamination. In their study, there was no increase in 95.8% (70/73) for 0.3μm particles and 94.5% (69/73) for 0.5 μm particles and no ATP contamination of the endoscopist’s gown and goggles in all cases. These results indicate that EB can prevent some exposure of endoscopists to aerosols and droplets and may be an effective option to minimize transmission. Thus, it remains important to develop devices that reduce patient suffering and improve endoscopist safety.

Kato et al. verified the usefulness of endoscopic ultrasound (EUS) with jelly-filling method for esophageal varices [8]. They retrospectively compared the quality of EUS images obtained by the water and jelly-filling methods. The EUS images were evaluated by two experts, and the EUS images obtained with the jelly-filling method were of significantly higher quality than those obtained with the water-filling method. The jelly-filling method could become the standard method for esophageal EUS. On the other hand, recently, viscous gel products have also been developed for securing the field of view during hemostasis of gastrointestinal bleeding [9,10]. The gel immersion method has the potential to be used for EUS as the jelly-filling method, and future research is expected.

Takasumi et al. compared the sample adequacy and diagnostic accuracy of endoscopic ultrasound fine needle biopsy (EUS-FNB) using the Fork-Tip Needle with endoscopic ultrasound fine needle aspiration (EUS-FNA) for gastric subepithelial lesions (SEL) [11]. In their study, the adequate sampling rate was 100% (13/13) for EUS-FNB and 90.9% (60/66) for EUS-FNA. The diagnostic accuracy was 92.3% (12/13) for EUS-FNB and 81.8% (54/66) for EUS-FNA, and was higher for EUS-FNB using a Fork-Tip needle, although the difference was not significant (*p* = 0.682). Although the histopathological diagnosis of gastric SEL is important in determining the treatment strategy, the accuracy rate of EUS-FNA for gastric SEL is not sufficiently high compared with that of EUS-FNA for pancreatic tumors [12,13,14]. Since immunohistochemistry and biomolecular tests are often required for the diagnosis of SEL, enough specimens obtained by EUS-FNB may have improved the diagnostic accuracy [15].

On the other hand, the remarkable development of gastrointestinal endoscopy in recent years is due to the development of IEE. In particular, the development and widespread of narrow band light observation, such as narrow band imaging (NBI) and blue light imaging (BLI), has dramatically improved the diagnostic accuracy of gastrointestinal tumors. Hatta et al. compared the diagnostic accuracy of magnifying endoscopy with BLI (ME-BLI) and NBI (ME-NBI) in determining the depth of invasion of superficial esophageal squamous cell carcinoma (SESCC) according to the intrapapillary capillary loop (IPCL) classification of the Japan Esophageal Society [16]. They registered ME-BLI and ME-NBI images of the same area of 81 SESCCs, and two blinded specialists diagnosed the depth of invasion from the images. In their study, there was no significant difference in the overall accuracy of invasion depth by IPCL classification between ME-BLI and ME-NBI (67.9–71.6% vs. 72.8–74.1%). The Kappa values for inter- and intra-observer agreement were also good. Ueda et al. also reported similar results in a retrospective study [17], and the diagnostic performance of ME-NBI and ME-BLI in diagnosing the depth of invasion of SESCC is expected to be comparable, but a prospective study with a larger number of cases is needed.

Meanwhile, new IEE have been developed. Texture and color enhancement imaging (TXI) is designed to enhance three image factors in WLI (texture, brightness, and color) to define subtle tissue differences clearly. TXI has two settings, mode1 with color enhancement and mode2 without color enhancement [18]. Dobashi et al. examined the color difference between the lesion and surrounding mucosa by WLI, TXI mode1, TXI mode2, and NBI in 59 SCC lesions of the pharynx and esophagus. The results showed that there was a significant color difference between the lesion and the surrounding mucosa in TXI mode1, TXI mode2, and NBI compared to WLI, and the color difference in TXI mode1 was the largest [19]. In addition, they evaluated the visibility of the lesions in each modality by three expert endoscopists and three non-expert endoscopists. The results showed that there was no difference in lesion visibility between expert and non-expert endoscopists in TXI mode 1, but in NBI, non-expert endoscopists were less effective in improving visibility by NBI than expert endoscopists.

Thus, a high level of expertise is required to fully demonstrate the power of image-enhanced observation, and in areas where expert endoscopists are not available, there are concerns about false negatives and misdiagnosis of lesions [20,21]. Kurumi et al. reviewed photodynamic diagnosis (PDD) for gastric cancer. PDD is an optical imaging technology based on the fundamental biological features of porphyrin metabolism in cancer cells and expected to be an objective endoscopic diagnostic method independent of expertise, for gastric cancer [22]. They showed the feasibility of PDD for gastric cancer, including their own experience.

In addition, one of the most rapidly evolving modalities in recent years is AI, which in recent studies has provided real-time diagnostics and has shown promising results in the first randomized trials against conventional endoscopic imaging. With the latest technological innovations, advances in endoscopic imaging are evident and have the potential to promote standardization of practice. Oka et al. gave a very detailed and clear review of the current status of AI, focusing on the areas of gastrointestinal disease, liver disease, and pancreatic disease [23].

As mentioned above, the field of upper gastrointestinal endoscopy is developing rapidly day by day. Endoscopists have a responsibility to constantly update their knowledge in the rapidly developing field of gastrointestinal endoscopy and apply it to their own daily practice.
